# Evolution and Structure of Proteins and Proteomes

**DOI:** 10.3390/genes9120583

**Published:** 2018-11-28

**Authors:** David A. Liberles, Ashley I. Teufel

**Affiliations:** 1 Department of Biology and Center for Computational Genetics and Genomics, Temple University, Philadelphia, PA 19122, USA; 2 Department of Integrative Biology, Institute for Cellular and Molecular Biology, and Center for Computational Biology and Bioinformatics, The University of Texas at Austin, Austin, TX 78712, USA

This themed issue centered on the evolution and structure of proteins and proteomes is comprised of seven published manuscripts. These works highlight recent developments in efforts to model protein evolution as well as findings from empirical computational genomes approaches. Two key inter-related themes emerge from the synthesis of these works: the crucial role of epistasis and the effect of mutation-selection-drift balance on protein stability.

Epistasis is suggested to have a significant impact on how proteins evolve [1,2,3,4,5], and several works within this themed issue explore the connection between epistasis and protein evolution. Posfai et al. [6] analytically demonstrate the necessity of epistasis in maintaining the stability of protein structures during evolution. Concrete examples of the significance of epistasis are also illustrated [7,8]. Knops et al. [7] describe how epistatic interactions in a Hepatitis C Virus protein can enable the evolution of drug resistance, while Andreou et al. [8] find that changes outside of the binding pocket of polysaccharide deacetylase in Bacillus species may contribute to differing substrate specificity. The need to account for epistatic interaction in evolutionary modeling is also pointed out in a review by Teufel et al. [9]. When modeling protein evolution with mutation-selection models, break points reflecting shifts in sets of appropriate amino acids can be used to maintain the mathematical approximation of site-independence while modeling a site-interdependent (epistatic) process.

The second theme that emerges from this collection of works is the role of mutation–selection–drift balance on protein stability, a concept popularized in a classic paper [10]. There are many sequence combinations that contribute to a folded state, and selection rarely acts on the precise energetic contributions of single amino acids, but rather on the cumulative effect in creating a properly folded protein (see reference [11] for further discussion of this point). This is what underlies epistasis. Ahrens et al. [12] look at the interplay of different structural features over evolution in maintaining folded states, while Mesa-Torres et al. [13] present examples in which disease arises due to the emergence of conformational instability with mutation that is not properly compensated. Further, Bányai et al. [14] demonstrate a correlation between rates of amino acid substitution, domain evolution, and morphological evolution in Cephalochordates by correcting mis-annotations that led to the inference of a higher rate of domain evolution in this lineage.

The link between epistasis and the site-specific substitution process emerged in the 1960s among other early work [15]. The combination of mutation at individual sites and selection at a higher level of organization reflecting fold and function gives rise to a clock-like rate of change. While this has subsequently been linked to the neutral theory, negative selection can also give rise to clock-like rates if substitutions are not rare and occur with a regular process (albeit made more complicated by epistasis). It is this understanding and its link to functioning under different selective regimes (including positive directional selection) that remain at the forefront of the field.

The exploration of epistasis within proteins and the effect of mutation–selection–drift balance on protein stability by the works compiled in this special edition are representative of the current state of research. Considering that these themes emerged organically from this compilation suggests that these areas of study pose many open questions. Each of these manuscripts represents a further step in developing this overarching understanding of the processes that govern molecular evolution at the protein level.
